# Fungal genomes mining to discover novel sterol esterases and lipases as catalysts

**DOI:** 10.1186/1471-2164-14-712

**Published:** 2013-10-18

**Authors:** Jorge Barriuso, Alicia Prieto, Maria Jesus Martínez

**Affiliations:** 1 Centro de Investigaciones Biológicas, Consejo Superior de Investigaciones Científicas, Ramiro de Maeztu 9, 28040 Madrid, Spain

**Keywords:** Hydrolases, Models, *In silico*, Fungal genomics, *Candida rugosa*, *Ophiostoma piceae*

## Abstract

**Background:**

Sterol esterases and lipases are enzymes able to efficiently catalyze synthesis and hydrolysis reactions of both sterol esters and triglycerides and due to their versatility could be widely used in different industrial applications. Lipases with this ability have been reported in the yeast *Candida rugosa* that secretes several extracellular enzymes with a high level of sequence identity, although different substrate specificity. This versatility has also been found in the sterol esterases from the ascomycetes *Ophiostoma piceae* and *Melanocarpus albomyces*.

**Results:**

In this work we present an *in silico* search of new sterol esterase and lipase sequences from the genomes of environmental fungi. The strategy followed included identification and search of conserved domains from these versatile enzymes, phylogenetic studies, sequence analysis and 3D modeling of the selected candidates.

**Conclusions:**

Six potential putative enzymes were selected and their kinetic properties and substrate selectivity are discussed on the basis of their similarity with previously characterized sterol esterases/lipases with known structures.

## Background

Lipases, also known as triacylglycerol lipases (EC. 3.1.1.3), act on ester bonds of several compounds, with acylglycerols as their natural substrates. These enzymes are able not only to catalyze under aqueous conditions the hydrolysis of triglycerides to free fatty acids, diglycerides and monoglycerides but also to carry out synthesis and trans- and interesterification reactions in the presence of organic solvents. Lipases are produced by animals, plants and microorganisms, but the latter have gained special interest in the industry due to their stability, selectivity or wide substrate specificity. Some of the most commercially important lipases belong to yeasts, such as *Candida rugosa* (synonym *C. cylindracea*) and *Candida antarctica*, or filamentous fungi, such as *Aspergillus niger*, *Humicola lanuginosa*, *Mucor miehei*, and *Rhizopus* species [1,2]. Sterol esterases (EC. 3.1.1.13), also known as cholesterol esterases, act on esters of sterols and long-chain fatty acids. The mammalian cholesterol esterases are the best known for their role in lipid metabolism and cholesterol absorption [3,4] but these proteins have also been reported in filamentous fungi, yeast and bacteria [5,6].

Both kinds of enzymes, lipases and sterol esterases, belong to the α/β-hydrolase superfamily and some of them show broad substrate specificity, including triglycerides and water insoluble sterol esters. For example, *C. rugosa* secretes a variety of closely related enzymes, commercialized as lipases or sterol esterases. Although at least three of them (Lip1, Lip2 and Lip3) display activity on both triglycerides and cholesteryl oleate, they differ in their specificity [7]. In the case of sterol esterases, this promiscuity has been reported for the enzymes characterized in *Ophiostoma piceae*[8] and *Melanocarpus albomyces*[9].

Lipases are generally strongly activated by water–lipid interfaces [10]. The active site of these enzymes presents a hydrophobic cavity covered by an amphipathic loop named “lid” or “flap” and most of them show interfacial activation, a phenomenon that occurs by the movement of the flap caused by a conformational change on this helical element, making the enzyme’s active site more accessible to the substrate [11]. This property has been considered as a distinguishing feature between lipases and sterol esterases although lipases lacking a flap [12] and sterol esterases showing interfacial activation have been reported [13,14]. These structural elements have been exhaustively studied in *C. rugosa* lipases and sterol esterases to understand the substrate specificity of the different isoenzymes. The five extracellular enzymes (Lip1–Lip5) characterized in this yeast have 534 amino acids, present a high level of sequence identity (77–88%) but show differences on pI and putative N-glycosilation sites [15]. The structural comparisons of three of them (Lip1, Lip2, and Lip3) revealed several amino acid changes affecting the flap, the substrate-binding pocket and the hydrophobic tunnel, that could be responsible for the differences in their catalytic properties [7]. This family of lipases forms the so called *C. rugosa* lipase-like family (abH03.01), which comprises proteins of relatively large molecular masses (>60 kDa) that contain a GESAG sequence located in the middle of the polypeptide chains, corresponding to the position 222 in *C. rugosa* Lip3, one of the enzymes described in more detail at the biochemical and structural levels [7]. The Ser in this sequence functions as a catalytic residue and constitutes a ‘catalytic triad’ together with the conserved Glu and His residues that are presumed to facilitate the hydrolysis [16]. These enzymes also contain the sequence GGGF involved in the oxyanionic hole (position 137 in *C. rugosa* Lip3), which allows the substrate entry into the catalytic pocket. Most of these characteristics are present in the *O. piceae* sterol esterase, which shows more than 40% sequence identity with *C. rugosa* lipases and similar substrate-binding sites, as suggested by its structural model based on the crystal structures of *C. rugosa* Lip3 [17].

Nowadays, with the development of new molecular techniques such as massive DNA sequencing, the genomes of an enormous number of organisms can be studied in a short time. In this sense, the Joint Genome Institute (JGI) from the US department of energy (DOE) was pioneer in this kind of projects, and more than 128 genomes from different fungi with potential biotechnological interest are currently accessible in its website (http://www.jgi.doe.gov/). Bioinformatics approaches to analyze these genomes allow the finding of new enzymes taking into account the analysis of conserved motifs in the available DNA sequences [18]. Furthermore, molecular modeling plays a key role in structural biology. Current methods to study protein structure are very interesting to discover enzymes with improved catalytic properties and activities [19].

In the present work we carried out a bioinformatics screening of public fungal genomes deposited at JGI to explore the presence of genes encoding sterol esterases/lipases from the genomes of environmental microorganisms, as a strategy to find novel enzymes. The candidates were selected taking into account the conserved motifs detected in versatile lipases and sterol esterases described in yeast or filamentous fungi. The kinetic properties of the new putative enzymes are discussed on the basis of their three-dimensional model structure, built from the crystal structures of *C. rugosa* lipases.

## Methods

### Genomes screening

Seven sterol esterases/lipases with wide substrate versatility and potential industrial application, as *C. rugosa* lipases (Lip1, 2, 3, 4 and 5) and sterol esterases from *O. piceae* (OPE) [8] and *M. albomyces*[9], were selected. The search for conserved motifs in the amino acidic sequence was carried out using MEME (http://meme.sdsc.edu/meme/intro.html). In all 7 sequences the motifs GESAG (forming the catalytic shoulder of the catalytic Ser) and GGGF (forming the oxyanionic hole) were found. These motifs are already described in the “The Lipase Engineering Database” for the lipases of the *C. rugosa* lipase-like family [20,21].

To search for genes codifying this kind of proteins in the public genomes from http://www.jgi.doe.gov/[22], the automatically predicted proteins from all 128 fungal genomes containing the terms “esterase” (4786 sequences) or “lipase” (6180 sequences) were downloaded using the Advanced Search option at the JGI web-site. From this pool of sequences (10976 redundant sequences) the ones containing the conserved motifs GESAG and GGGF were selected using the Bioedit 7.1.3 software.

### Phylogenetic analysis

**Figure 1:**
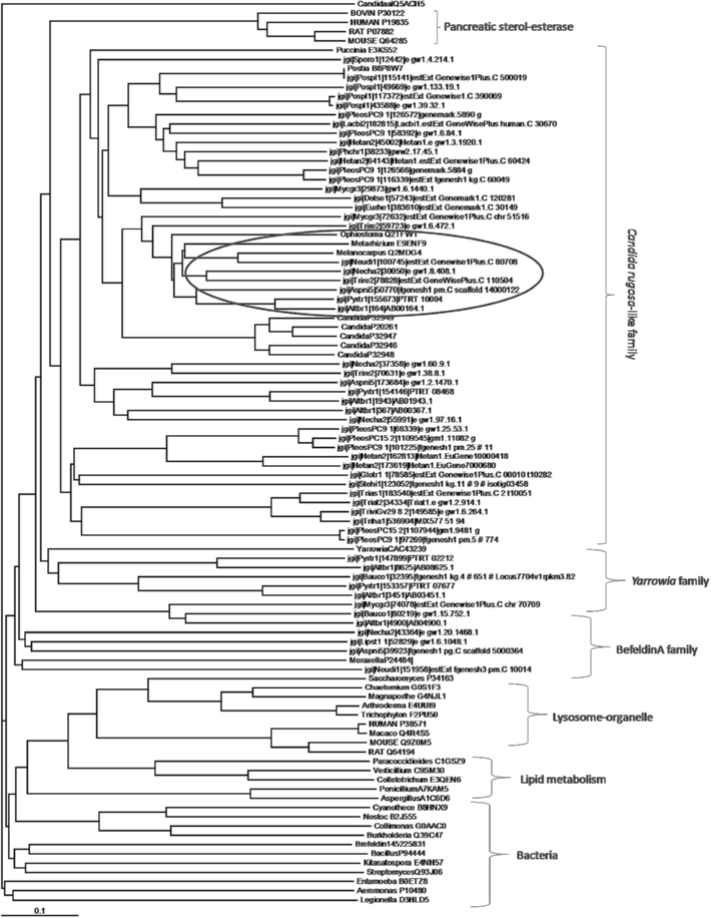
**Phylogenetic tree of putative protein sequences of fungal sterol esterase/lipases.** Phylogenetic tree of 56 putative protein sequences of sterol esterase/lipases from fungal genomes, and representative sequences of the *C. rugosa*-like family, Y*arrowia* family, *Brefeldin* family, cellular organelles, pancreatic sterol esterases, and bacterial esterases. The 6 selected candidates are marked with an oval.

A phylogenetic tree was built using the 56 protein sequences selected in the previous section and representative sequences of different lipase and sterol esterase families: *C. rugosa*-like (ascomycetes and basidiomycetes), *Yarrowia* family, brefeldin family, cellular organelles, pancreas and bacteria (accession numbers in Figure 1). An un-rooted tree was created using MUSCLE multiple sequence alignment and the Maximum-Likelihood method. Treeview 1.6.6 was used to draw the trees. Sequences grouping with the versatile lipases and sterol esterases from the *C. rugosa*-like family were selected for further analyses.

### Sequence analysis

Sequences from the 6 candidates selected in the previous section were compared by means of BLAST against the NR (NCBI) and “The Lipase Engineering Data Project” (LED) databases. Sequence alignments were also carried out versus *C. rugosa* Lip3 sequence using Clustal X 1.8, and putative signal peptides in each sequence were predicted using SignalP 4.0 [23].

### Structural models

A three-dimensional model of the selected putative proteins was generated using the programs implemented by the automated protein homology-modeling server SWISS-MODEL (Swiss Institute of Bioinformatics [24]). In all cases the selected template, with higher homology to the putative sequence, was C. rugosa Lip3 (Parent PDB 1llf, Chain B). The 6 models matched all the sequence length excepting the putative signal peptide.

The models were exhaustively analyzed using PyMol 1.1 (http://pymol.org/). Putative intramolecular tunnels were modeled from the catalytic serine using Caver 2.0 v0.003 [25].

## Results and discussion

This study shows an interesting bioinformatics approach to find new sterol esterases/lipases from the genomes of environmental microorganisms thanks to the natural engineering process driven by evolution.

### Identification of conserved motifs in lipase/sterol esterase enzymes and search in public microbial genomes

After the analysis of the protein sequences described as lipase/sterol-esterase with versatile activity against triglycerides and sterol esters, two conserved motifs described for α/β hydrolases of the *C. rugosa*-like family were identified: GGGF and GESAG.

The search of putative proteins automatically annotated as sterol esterase or lipase in the 128 public genomes deposited in the JGI returned only 56 sequences from 26 different genomes.

### Phylogenetic analysis

Figure 1 shows a phylogenetic tree with the 56 putative enzymes from the different genomes analyzed, as well as representative sterol-esterases/lipases from different fungal families. Twelve putative proteins from the JGI genomes grouped with the lipases from the lipolitic yeast *Yarrowia*[26] or the *Bacillus subtilis* Brefeldin A family [27], typical enzymes with high affinity for triglycerides whose ability to hydrolyze sterol esters has not been reported. On the other hand, this figure shows 44 of the candidate proteins grouped with the *C. rugosa*-like family, and 6 out of these putative proteins were selected to continue the study because they were the most closely related to the proteins from *C. rugosa* (P20261, P32946, P32947, P32948 and P32949) [7,15], *O. piceae* (Q2TFW1) [8], and *M. albomyces*[9] with activity on both triglycerides and sterol esters. The 6 candidate proteins belong to different organisms but all of them are plant-associated fungi (Table 1). Species from the genus *Neurospora* are known for their ability to colonize the wood from the trees after forest fires, [28]. The plant pathogenic *Nectria haematococca* has also been related to the degradation of aromatic recalcitrant compounds, [29,30]. *Trichoderma* species are frequently isolated from forest or agricultural soils, and are known for their ability to produce enzymes of industrial relevance [31]. *Aspergillus niger* secretes a wide array of hydrolytic and oxidative enzymes [32]. *Pyrenophora tritici-repenti* is a plant pathogen causing a disease called “tan spot” that mainly affects wheat [33]. Finally, the genus *Alternaria* comprises many common saprophytic and plant pathogenic species [34].

**Table 1:** Candidate sequence analysis

**Selected candidates (putative protein JGI accession number)**	**Fungal species**	**CDS length**	**Homology against NCBI**	**Homology against LED**	**% of similitude with Lip3**
jgi|Neudi1|100745|	*Neurospora discreta*	1737 nt	75% with Q2MDG4	75%with Q2MDG4	42%
jgi|Necha2|30050|	*Nectria haematococca*	1764 nt	64% with Q2MDG4	62% with Q2MDG4	39%
jgi|Trire2|78828|	*Trichoderma reesei*	1713 nt	59% with Q2MDG4	59%with Q2MDG4	40%
jgi|Aspni5|50770|	*Aspergillus niger*	1689 nt	64% with Q5XTQ4	57% with Q5XTQ4	40%
jgi|Pyrtr1|155673|	*Pyrenophora tritici-repentis*	1683 nt	62% with Q5XTQ4	62% with Q5XTQ4	46%
jgi|Altbr1|164|AB00164	*Alternaria brassicicola*	1686 nt	64% with Q5XTQ4	61% with Q5XTQ4	44%

Accession number Q2MDG4 corresponds to sterol esterase precursor from *Melanocarpus albomyces*, and accession number Q5XTQ4 to *Botryotinia fuckeliana* lipase.

The ecological role of the extracellular lipases/esterases in these fungi can be related to the initial degradation of the epicuticular waxes and cuticle, which consist of a mixture of long-chain fatty acids, aldehydes, alkanes, primary and secondary alcohols, ketones and wax esters [35]. The deterioration of these external layers makes the plant cell-wall polysaccharides more accessible as carbon source for the plant-associated fungi, or facilitates the colonization by pathogenic species [36,37].

### Candidate sequences analysis

The six candidate sequences as putative versatile enzymes with lipase and sterol esterase activity were compared against NCBI and LED databases using Blastp. In all cases, the highest score matched with both a sterol esterase precursor from *M. albomyces*[9] and the lipase from *Botryotinia fuckeliana*[38]*,* with sequence identities between 57 and 75% (Table 1). The sequence identity of each candidate with the *C. rugosa* Lip3, used as a model protein, is shown in Table 1. All these data correlate with the results from the phylogenetic analysis (Figure 1).

Additional file: 1 Figure S1 shows the sequence alignment of Lip3 against the versatile lipase/esterase candidates, highlighting the predicted signal peptides at the N-terminal of the putative proteins consisting in 15 to 22 amino acids, the 2 conserved Cys residues forming disulfide bonds and delimiting the lid region, the oxyanionic hole (GGGF), and the catalytic triad (Ser-Gln-His). An N-glycosylation site is present in all the sequences analyzed, except for jgi|Trire2|78828|.

Moreover, it is known that the number of hydrophobic amino acids at the lid region is related to enzyme-substrate specificity [39]. Lipase isoenzymes from *C. rugosa* share over 80% sequence identity but diverge in the sequence of the lid. In the active enzyme conformation, the open lid participates in the substrate-binding site and contributes to substrate recognition. The substitution of the lid from isoenzyme Lip1 for that of Lip3 was sufficient to confer to Lip1 cholesterol esterase activity [39]. Additional file: 1 Figure S1 shows the differences in the residues located at the lid region of the putative proteins. The number of hydrophobic residues is 14 in Necha2 and Trire2, 13 in Altbr1, 12 in Pyrtr1 and Neudi1, and 11 in Aspni5, while Lip3 only contains 10. The number of hydrophobic residues in the lid of the different lipases produced by *C. rugosa* is higher in the isoforms that are more active on sterol esters than on triglycerides: Lip2 > Lip3 > Lip1 (12, 10, and 8 hydrophobic residues, respectively) [7,15]. According to this data, the *O. piceae* sterol esterase, with 13 hydrophobic residues in lid region, has been reported to possess higher activity on sterol esters than *C. rugosa* Lip3 [17]. Based on these observations it can be inferred that Necha2, Trire2, Pyrtr1, and Altbr1 may have high affinity for sterol esters, while Neudi1 and Aspni5 could have intermediate characteristics.

### Molecular modeling analysis of selected candidates

**Figure 2:**
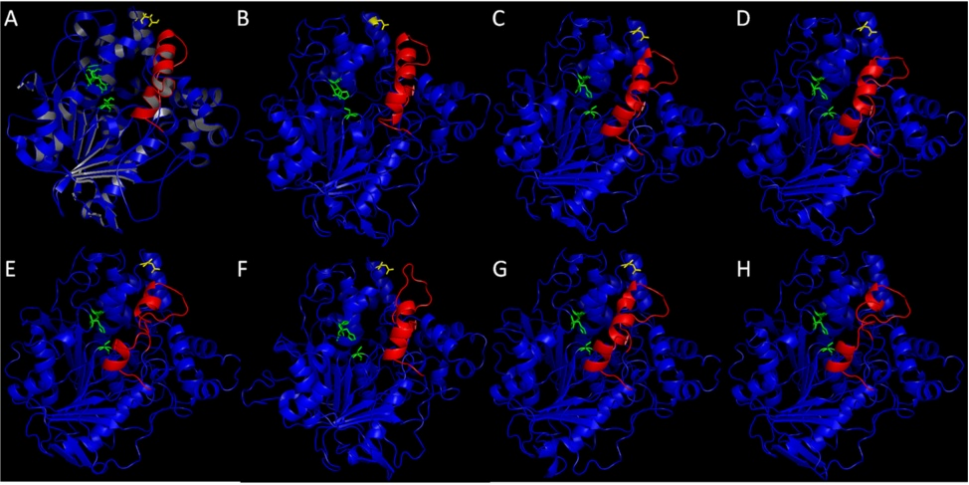
**Tridimensional structures/models of the proteins in its open conformation. (A)** Lip3 from *C. rugosa*. Models, based in *C. rugosa* Lip3 structure, from **(B)** OPE and the six selected putative proteins **(C)** Altbr1, **(D)** Aspni5, **(E)** Necha2, **(F)** Neudi1, **(G)** Pyrtr1 and **(H)** Trire2. Lid region is indicated in red, the three catalytic residues are highlighted in green and the residue for a putative glycosylation site is marked in yellow.

Molecular models of the selected putative candidate sequences were generated using SWISS-MODEL server and in all cases the template automatically selected was Lip3 from *C. rugosa* (pdb 1llfB and 1cle), one of the versatile lipases better characterized [5-7]. Figure 2 displays the comparison of the 3D structures obtained, showing the typical α/β-hydrolase conformation [40] with a lid region formed by an α-helix which, in most lipases, allows a conformational change in the presence of the substrate improving its accessibility to the active site [11]. A superimposition of the residues from the catalytic triad in the 6 candidate models and the Lip3 structure, showed an identical geometry in all cases, with the catalytic Ser close to the mouth of the substrate pocket (data not shown).

The percentage of hydrophobic residues at a distance of 4 Å or shorter from the predicted tunnels was calculated for each model. Trire2 had 11 hydrophobic residues out of 22 in the tunnel area (50%), similar to Aspni5 that had 9 out of 18 (50%) and Pyrtr1, 11 out of 21 (52%). However, Neudi1 presented a higher number of these residues 11 out of 20 (55%) and Necha2 showed even higher number, 13 out of 22 (59%). Altbr1 had less hydrophobic residues 8 out of 22 (36%). In the case of *C. rugosa* lipases, Lip1 had 9 out of 21 (42%), Lip2 presented 12 out of 25 (48%) and Lip3 showed 11 out of 24 (46%), being Lip2 and 3 the ones with the highest affinity for sterol esters. OPE, with higher affinity than any Lip on sterol esters, has 10 hydrophobic residues out of 21 (48%) in the tunnels area. Since all the selected putative enzymes, except Altbr1, presented more hydrophobic residues than OPE and Lip2 and 3, this could be indicative of its increased affinity for sterol esters.

**Figure 3:**
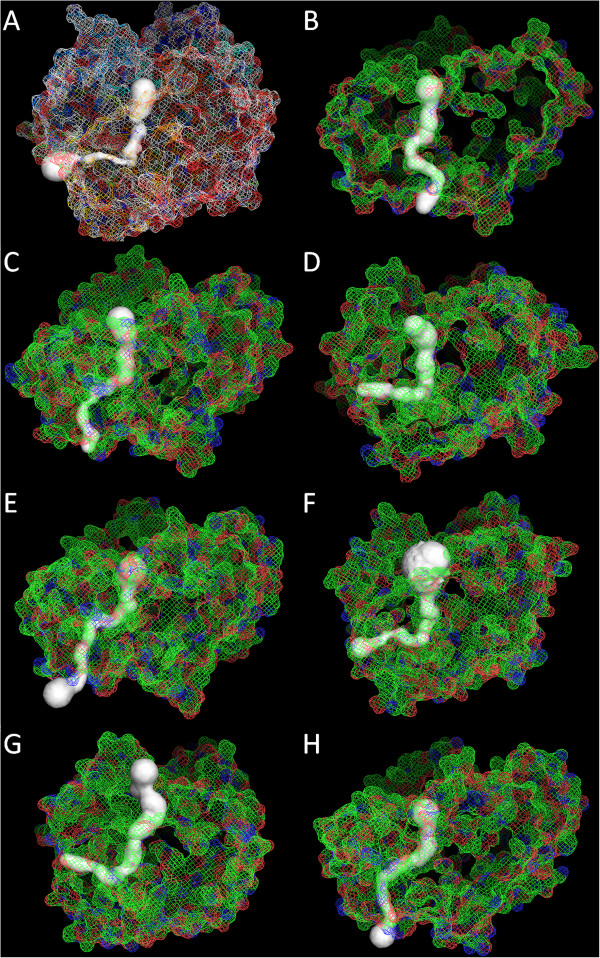
**Models of the intramolecular tunnels.** Internal tunnels in each structure were modeled using Caver 2.0. **(A)** Lip3 (model structure: PDB 1llf, Chain **B**), **(B)** OPE, **(C)** Altbr1, **(D)** Aspni5, **(E)** Necha2, **(F)** Neudi1, **(G)** Pyrtr1 and **(H)** Trire2.

The model of the internal tunnels in each structure was made using Caver 2.0 (Figure 3). In all cases, a big tunnel coinciding with the substrate binding pocket and a second putative tunnel that may be an exit for the release of the reaction products can be observed. This hypothesis was already proposed for *C. rugosa* Lip1 as a possible mechanism responsible for the high catalytic efficiency of this enzyme [41]. The length and shape of these tunnels differ for each model and the residues forming the tunnels can also vary. Lip3 has a prominent entry tunnel and a bended exit tunnel, similar to that reported for Lip1 [41], while the *O. piceae* sterol esterase model presented a broad straight tunnel opposed to the substrate pocket, which could contribute to its reported higher catalytic efficiency as compared with Lip3 [17].

Neudi1, Pyrt1 and Aspni5 showed a prominent entry tunnel and a bended exit tunnel, similar to Lip3 structure (Figure 3A, D, F and G). Altbr1, Necha2 and Trire2 presented a broad straight tunnel opposed to the substrate pocket, in this case more similar to that found in *O. piceae* sterol esterase (Figure 3B, C, E and H). As mentioned before, Altbr1 has the smallest hydrophobic residues content in the tunnel, indicating that this characteristic is not strictly related to its shape, and that it could be a very efficient enzyme oxidizing triglycerides. All these data suggest that those putative enzymes with OPE-like tunnels could be more efficient than those with *C. rugosa*-like tunnels, allowing a faster entry of the substrate and exit of the products, and the hydrolysis of big substrates.

## Conclusions

Here we present a useful strategy to identify enzymes with potential biotechnological interest by means of genomes mining. The strategy combines search of conserved motifs, phylogenetic analysis, sequence homology studies and protein model analysis. This strategy allowed identifying 6 candidate sequences (Table 1). The six putative proteins selected were automatically annotated at JGI server except for Aspni5 that has been manually annotated as a putative sterol esterase with homology to the enzyme from *O. piceae*. In this study we inferred the potential characteristics of the putative enzymes based on their model structures. Our analysis, derived from the percentage of hydrophobic residues in the lid region, suggests that the affinity towards sterol esters of all the candidate proteins selected could be superior to that of the *C. rugosa* model lipases. In addition, the putative proteins Altbr1, Necha2, and Trire2 may also have higher efficiency against this substrate than *C. rugosa* lipases due to their exit tunnel conformation. However, further studies on the expression and characterization of these new proteins will be necessary to corroborate their potential biotechnological or industrial interest.

## Competing interests

The authors declare that they have no competing interests.

## Authors’ contributions

JB participated in the overall conceptual and experimental design of this study, analysis and interpretation of results, as well as in drafting the manuscript. AP participated in its design and coordination and helped to draft the manuscript. MJM participated in the analysis and interpretation of results, and in drafting and revising the manuscript. All authors read and approved the final manuscript.

## Supplementary Material

Additional file 1: Figure S1 Sequence alignment of the putative proteins against Lip3. Sequence alignment of model protein Lip3 from *C. rugosa* against the six putative sterol esterase/lipases selected from fungal genomes. The lid region is written in red. The predicted signal peptides are highlighted in green, cysteines in blue, the oxyanionic hole in yellow, the catalytic triad residues (Ser-Gln-His) are underlined and written in orange and the conserved surrounding residues are underlined. A putative N-glycosylation site is highlighted in magenta. Click here for file
