# Supplementary figures and images for: Fungal genomes mining to discover novel sterol esterases and lipases as catalysts
Source: BMC Genomics. 2013 Oct 18;14:712. doi: 10.1186/1471-2164-14-712 (PMC3827930; doi:10.1186/1471-2164-14-712)

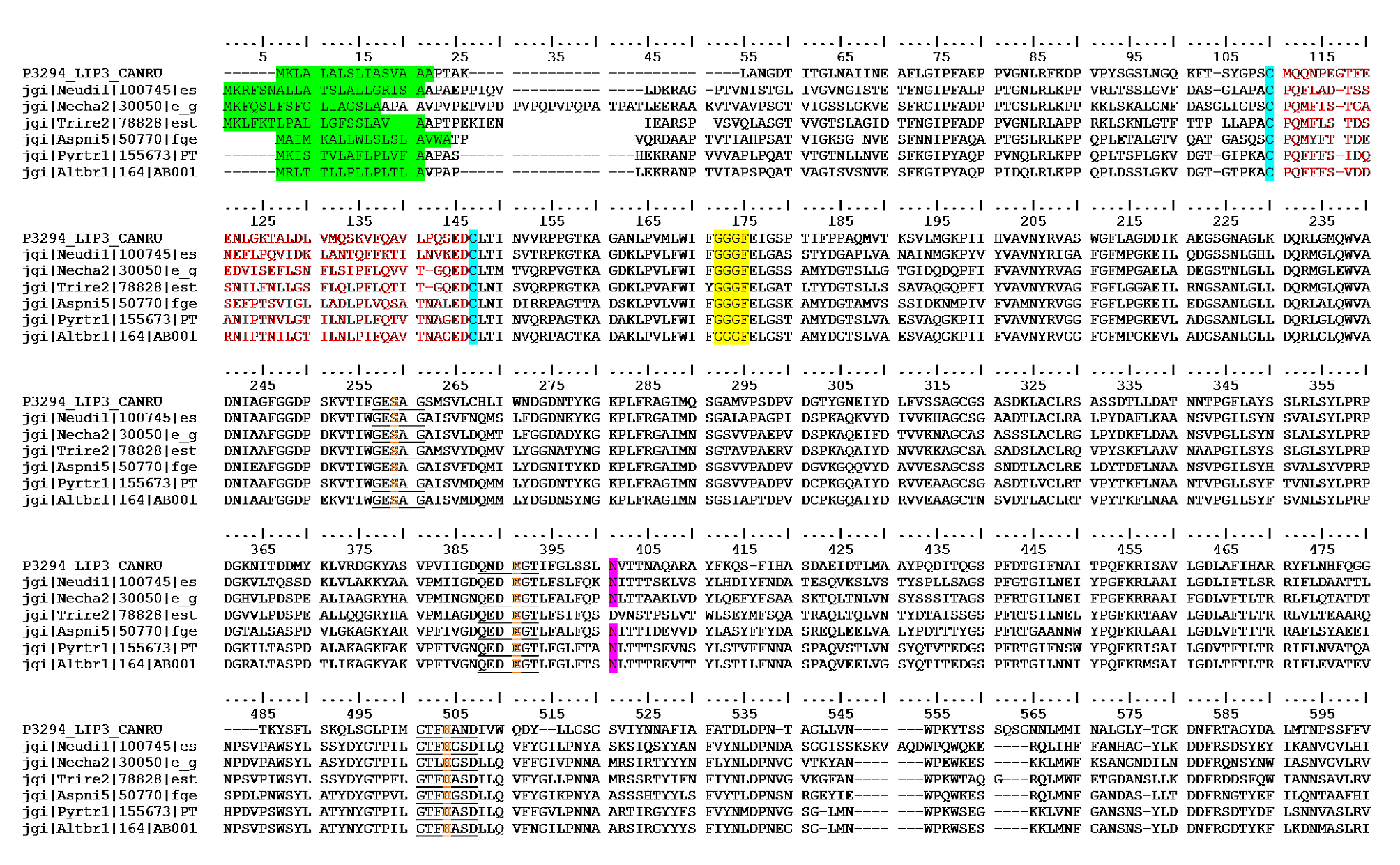

Supplement: Additional file 1: Figure S1 — Sequence alignment of the putative proteins against Lip3. Sequence alignment of model protein Lip3 from C. rugosa against the six putative sterol esterase/lipases selected from fungal genomes. The lid region is written in red. The predicted signal peptides are highlighted in green, cysteines in blue, the oxyanionic hole in yellow, the catalytic triad residues (Ser-Gln-His) are underlined and written in orange and the conserved surrounding residues are underlined. A putative N-glycosylation site is highlighted in magenta. [file 1471-2164-14-712-S1.tiff]
